# The role of Community Health Workers in supporting caregivers of Hispanic/Latino older adults with dementia and the need for workforce expansion

**DOI:** 10.1002/alz.087664

**Published:** 2025-01-09

**Authors:** Wesli H Turner, Shannon Patrick, Saúl Juárez Aguilar, Maria C Mora Pinzon

**Affiliations:** ^1^ MHP Salud, Ypsilanti, MI USA; ^2^ ProHealth Care, Waukesha, WI USA; ^3^ Wisconsin Alzheimer’s Institute, University of Wisconsin ‐ Madison, Madison, WI USA

## Abstract

Community Health Workers (CHWs), also known as *Promotores de Salud* expand health equity to underserved and hard‐to‐reach communities by providing increased access to healthcare and support services for caregivers and persons living with dementia (PLWD). CHWs have been particularly useful in supporting Hispanic/Latino communities, who are 1.5 times more likely to develop Alzheimer’s Disease and related dementias than non‐Hispanic whites and are less likely to access support services due to language and cultural barriers. CHWs offer support in culturally appropriate ways, linking families to much needed services and assistance acting as a crucial support system for many who lack the knowledge of where to access services or face cultural or linguistic barriers to accessing those services. When CHWs are integrated into care teams, they can reduce the burden on other health professionals by focusing on addressing the social determinants of health, allowing health professionals to focus on their area of expertise with patients. CHWs can increase awareness of the disease, promote activities that reduce the risk factors for ADRD, provide navigation services, and facilitate caregiver support groups.

This presentation will highlight the value of CHWs in providing culturally and linguistically appropriate dementia caregiver support within Hispanic/Latino communities. Presenters will discuss outcomes from a CHW‐led caregiver support program that showed a 22% increase in knowledge of ADRD (p <0.001), 56% increase in caregiver preparedness skills (p <0.001) and showed a 33% reduction in depression symptoms among participants (p <0.001). To date, 82 caregivers have been served through this program, supporting the development of the CHW workforce in assisting caregivers and PLWD.

Presenters will also detail a novel HRSA‐funded CHW training program that creates a workforce pipeline from the community to the healthcare system providing paid training through a federal grant. From March 2023 to January 2024, 105 CHWs have been trained across 17 counties in Florida, of which 58% identify as Hispanic/Latino. By demonstrating the possibilities of a workforce pipeline, this presentation will provide the “How” and “What” of bridging the growing labor gap in supportive services for Hispanic/Latino persons living with dementia and their caregivers using CHWs.

